# Rapid Onset Chylous Ascites Presenting as the Initial Manifestation of Follicular Lymphoma: A Case Report

**DOI:** 10.7759/cureus.27199

**Published:** 2022-07-24

**Authors:** Benjamin Weber, Nicholas D Luke

**Affiliations:** 1 Hematology and Oncology, St. Michael Medical Center, Newark, USA; 2 School of Medicine, St. George's University School of Medicine, True Blue, GRD

**Keywords:** paracentesis, rchop therapy, lymphadenopathy, lymphoma, ascites

## Abstract

Follicular lymphoma is the most common indolent Non-Hodgkin Lymphoma (NHL) with overall survival measured in years for the majority of patients (NHL carries a somewhat positive prognosis). Baseline clinical genetic characteristics appear to be the best predictors of the clinical course. A few cases appear to be familial; however, no consistent environmental or infectious causation has been identified. Our presenting patient has a case of follicular lymphoma that was initially diagnosed in the setting of rapid onset chylous ascites, a highly atypical and rare presentation. Follicular lymphoma typically presents with painless lymphadenopathy in lymph nodes along the torso. The lymphoma cells divide within the nodes and can be palpated as bumps within the axilla or on the neck above the clavicle. Various prognostic indicators and tumor grading can give providers a sense of survival statistics. Once diagnosed, chemotherapy tends to form the backbone of most treatment regimens with various roles for immunotherapy and radiation.

## Introduction

Follicular lymphoma (FL) is among the most common subtypes of non-Hodgkin lymphomas (NHLs) and typically follows an indolent course. It may account for as much as 35% of NHLs in the United States by current estimates. Absolute risk factors have not been definitively confirmed; Familial risk, environmental exposure, infection, and others have been theorized. One rare aspect of follicular lymphoma that can occur is when it presents with chylous ascites. Chylous ascitic fluid is an uncommon characteristic, which is seen as a milky-white colored fluid and an elevated triglyceride level with chylomicrons within the ascitic fluid [1]. Current scientific models propose FL stems from germinal center B cells between centroblasts and centrocytes [2]. Overexpression of BCL2 oncogene along with (14;18) translocation and immunoglobulin-mediated B cell receptor signaling have been observed [3]. Excisional tissue/nodal biopsy can best confirm the diagnosis. Once the diagnosis has been made, scoring by follicular lymphoma IPI (FLIPI), PRIMA prognostic Index (PRIMA-PI), along with grading/staging provide a sense of overall prognosis [4]. Treatment modalities may range from radiation therapy (RT) to immunotherapy, chemoimmunotherapy, or a combination of methods. The approach to FL treatment depends on whether a patient meets the Groupe d'Etude des Lymphomes Folliculaires (GELF) criteria or not. The criteria take into consideration the number of enlarged lymph nodes and their size(s), systemic symptoms, symptomatic organ compression, splenomegaly, bone marrow infiltration, and the presence of cytopenia. While early treatment may not improve overall survival, the progression-free survival, or the length of time in which the patient does not experience worsening symptoms during or after treatment, does improve with an early start in treatment for follicular lymphoma. Treatment response may be variable, and the dose of radiation given to one patient may differ drastically from another [5]. Aside from radiation and chemotherapy, immunotherapy such as rituximab can be given either through an intravenous or intraperitoneal route in the setting of ascites secondary due to follicular lymphoma [6]. One of the best predictors of overall survival is based on the treatment response and disease progression at 24 months. Another factor to consider is that the stage of the follicular lymphoma may cause the treatment response to suffer and be less efficacious. A stage three or four follicular lymphoma will not see improvements in overall survival or progression-free survival in comparison to a stage one or two. In this case report, we present a case of follicular lymphoma with an initial presentation of rapid onset of chylous ascites.

## Case presentation

Our presenting patient is a 69-year-old female with a past medical history of brain cancer status post resection, seizure disorder, hypertension, gastroesophageal reflux disease (GERD), and previous COVID-19 infection. She presented to the emergency department (ED) with a rapid weight gain of seven pounds in three days, along with abdominal distention. She denied any associated pain or other complaints at the time of admission.

**Figure 1:**
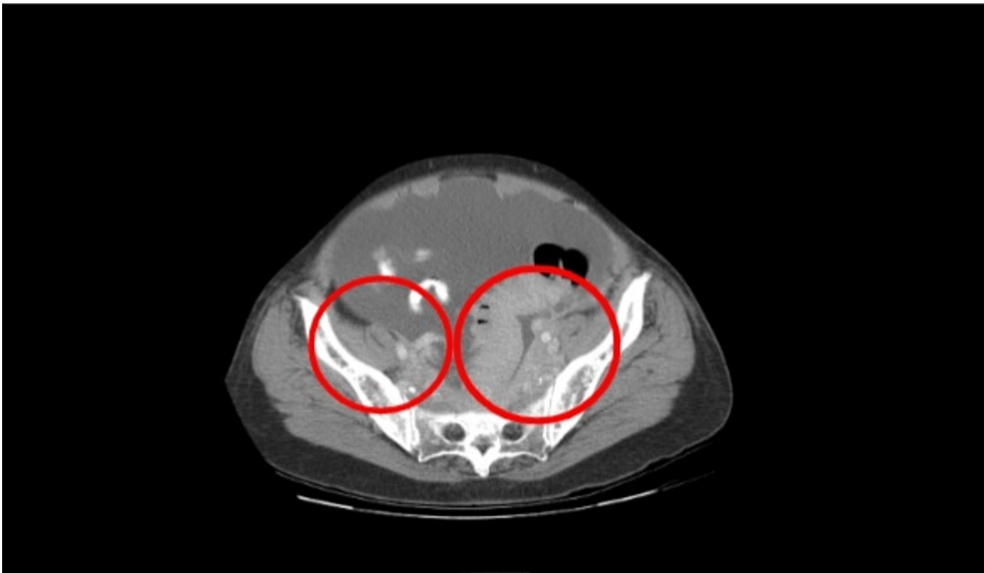
CT abdomen/pelvis with oral and IV contrast Extensive soft tissue masses encasing the mesenteric vessels, omentum, retroperitoneal, and sigmoid colon and rectum (red circles)

**Figure 2:**
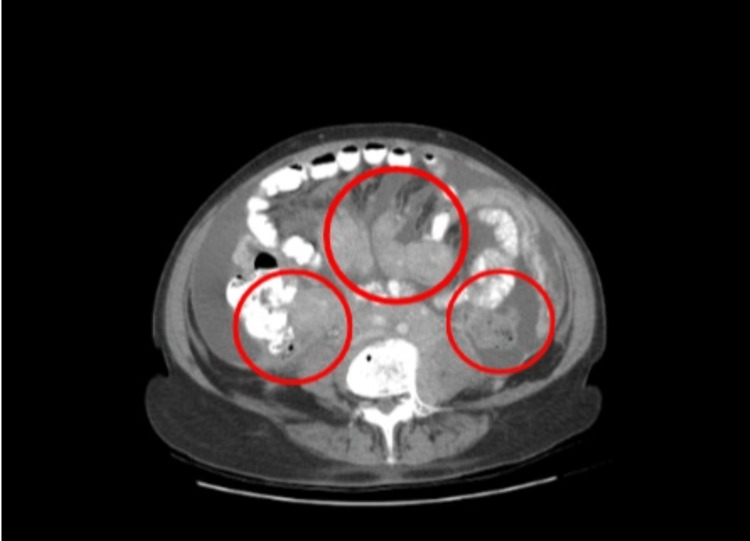
CT abdomen/pelvis with oral and IV contrast Extensive soft tissue masses encasing the mesenteric vessels, omentum, retroperitoneal, and the sigmoid colon and rectum (red circles)

On admission, her temperature was 97 degrees Fahrenheit, her blood pressure was 135/69 mmHg, her heart rate was 98 beats per minute, 16 respirations per minute, and saturating 98% oxygen on room air. On exam, she was alert, oriented, and in no distress. The cardiopulmonary exam was within normal limits. An abdominal exam did reveal a hard, distended, non-tender abdomen with a positive fluid wave, and normal bowel sounds. Significant labs on admission included a white blood cell count (WBC) of 4.0 x 10^3/mcL and a hemoglobin of 11.9 g/dL. A computed tomography scan of the abdomen and pelvis with oral and intravenous (IV) contrast demonstrated extensive soft tissue masses encasing the mesenteric vessels, omentum, retroperitoneal, and sigmoid colon and rectum (Figures 1-2). There was also infiltration of the bilateral renal pelvis with prominent ascites.

**Figure 3:**
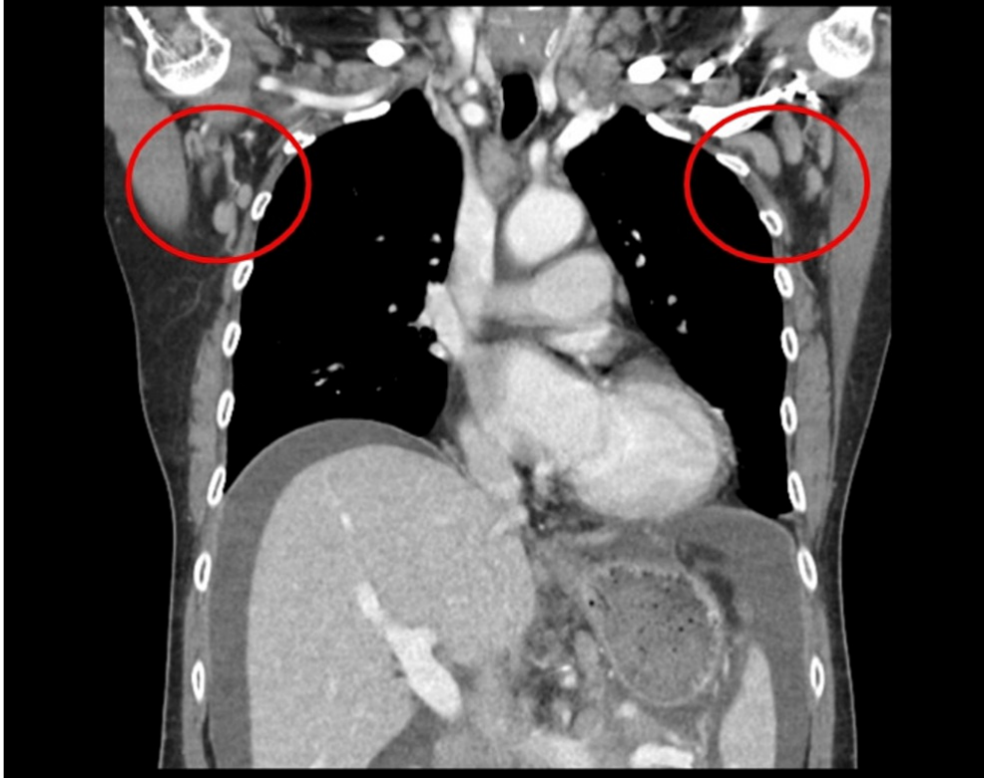
CT Chest with Contrast 2/9 showing bilateral axillary adenopathy (Red circles).

**Figure 4:**
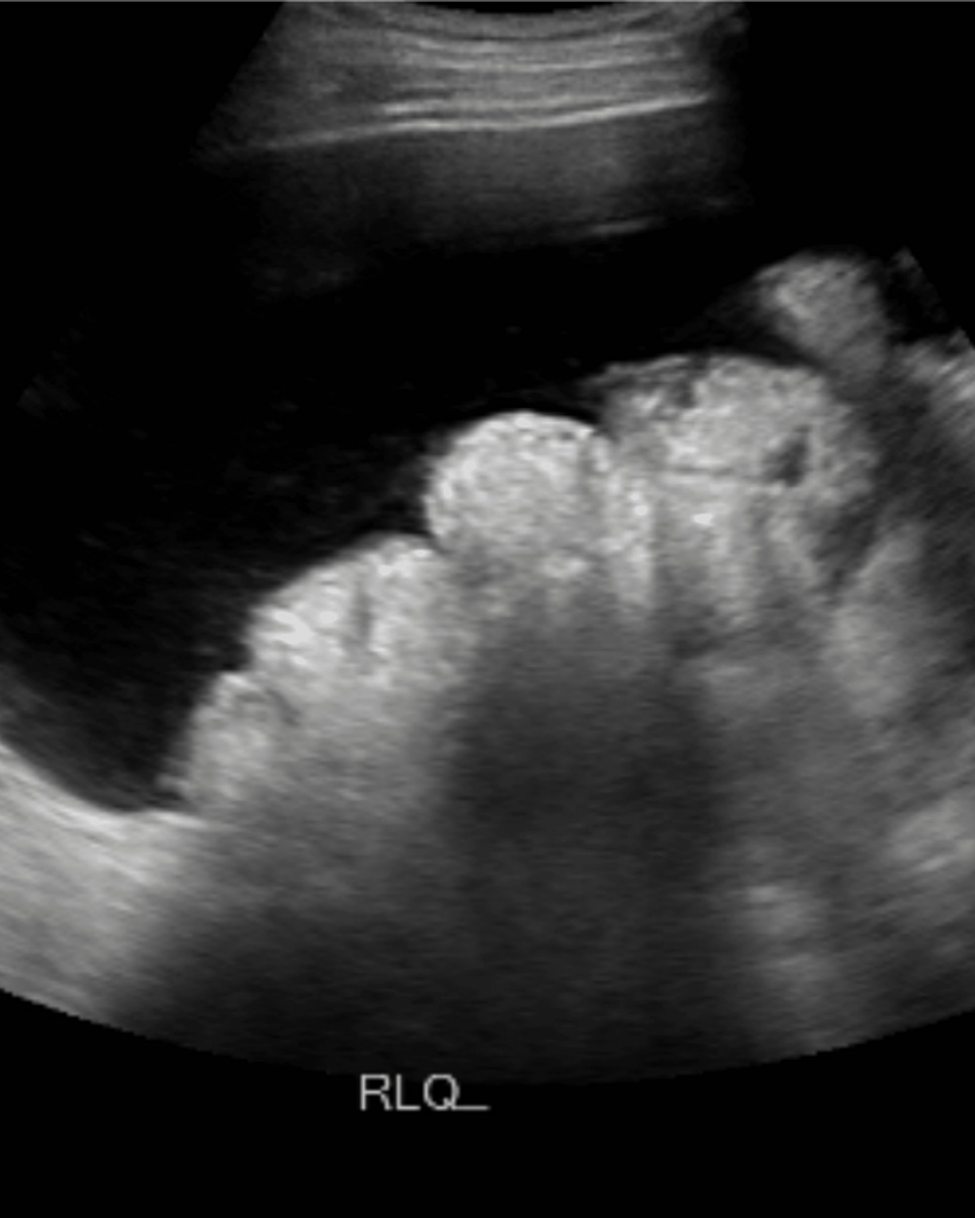
Ultrasound Paracentesis finding of a total of 5500cc of chylous ascites prior to removal.

CT of the chest with contrast was ordered to evaluate for metastasis; bilateral axillary lymphadenopathy was noted (Figure 3). Ultrasound-guided paracentesis was performed for the suspected malignancy: 5500 mL of milky-white ascitic fluid was drained (Figure 4). The triglyceride level was 993 mg/dl. Cytology was negative for malignant cells and included reactive mesothelial cells, many lymphocytes, and scant macrophages. Carcinoembryonic antigen (CEA) and cancer antigen 125 (CA125) were found to be 0.3 ng/mL and >1000 U/mL, respectively. There was no mediastinal or hilar adenopathy, no lung infiltrates, free fluid, or pleural thickening at the apices and bases. An excisional biopsy of the right cervical lymph nodes showed follicular lymphoma, WHO grade 2/3 with Ki-67 proliferation index high at 70%. Bone marrow biopsy was performed, which showed normocellular marrow with involvement (5-10% cellularity) by follicular lymphoma. CD20 showed paratrabecular B cell infiltration with coexpression of CD10, BCL6, and BCL2. The Ki67 proliferation index of B cells was low, at <5%. A repeat paracentesis on days four and eight of admission in light of the re-accumulation of ascites along with the insertion of a port-a-cath for chemotherapy was done. Upon performing the third paracentesis, the patient was deemed stable for discharge, and arrangements were made for the patient to follow up with the oncology clinic.

The paracentesis panel demonstrated no organisms on gram stain, a pH of 8.0, lactate dehydrogenase (LDH) of 94 IU/L, a triglyceride level of 993 mg/dL, a glucose level of 129 mg/dL, a cholesterol level of <50 mg/dl, an amylase level of 33 U/L, and an albumin fluid level of 2.2 g/dL. A fluorescence in situ hybridization (FISH) study was performed and was positive for a rearrangement involving BCL2 (45% of cells), and negative for MYC and BCL6 rearrangements. Flow cytometry showed a monoclonal lambda B cell population (86% of the total cells), these cells expressed CD10, CD19, CD20, CD22, CD23, CD38, and CD71. Normal T cells compromised 12% of total cells.

## Discussion

This case outlines a highly atypical presentation of follicular lymphoma with an initial onset of rapid chylous ascites. In unusual circumstances such as this, the malignancy can disrupt the subserosal lymphatic flow, which may lead to the accumulation of chylous fluid in the abdominal cavity. This loss of proteinaceous material can predispose patients to infection and generalized organ dysfunction. In addition, this leakage of viscous contents into the relatively fixed abdominal space has been linked to the development of abdominal compartment syndrome, a critical diagnosis [7-8]. Although our presenting patient did not develop abdominal compartment syndrome, the rapid rate of fluid accumulation warrants a high index of suspicion for such serious complications. If treating a patient with underlying liver pathology, ascites may be mistaken with cirrhotic fluid. Such ascites can be treated with paracentesis and radioimmunotherapy such as R-CHOP (rituximab-cyclophosphamide-hydroxydaunorubicin-Oncovin-prednisone regimen) [4-6]. In our presenting patient, the Ki-67 index was 70%, which means that the malignancy can potentially undergo Richter's transformation and a positron emission tomography (PET) scan, excisional biopsy, or an image-guided core needle biopsy of an enlarged lymph node should have been performed in retrospect. Liu presents a case in which the fluid’s gross appearance and serum ascites albumin gradient (SAAG) value of greater than 1.1 g/dL typically align with that of portal hypertension [9]. Other causes must be ruled out such as tuberculosis (TB), hepatitis, vasculitis, rheumatologic causes, and cardiac and renal etiologies. In light of the wide differential, some practitioners have even stumbled upon a follicular lymphoma diagnosis after extensive workup requiring the use of laparoscopy [8].

As for the approach to therapy, Taveres touches on the potential for radiotherapy (24-30 Gy for low-grade, early-stage, and higher doses if more advanced) in conjunction with rituximab [4-5]. Jagosky illustrates how modern regimens tailored to particular lymphoma subtypes have drastically improved outcomes - prior to 1982, chylous ascites previously marked a greater than 90% mortality merely three months after diagnosis [2]. During the course of treatment, another major consideration is the possible transformation into diffuse large B cell lymphoma (DLBCL). In one example, Babu presents a case that transforms to DLBCL and then back to FL. A case may be possible due to treating the aggressive component of the lymphoma while the relapse occurs afterward if the indolent aspect of the lymphoma remains. Low albumin, high FLIPI score, grade 3+, high LDH, and presence of B symptoms were among the major red flag characteristics associated with a higher risk of transformation while the use of rituximab was correlated with a lower transformation rate [4,6,10].

Follicular lymphoma can be treated with R-CHOP (rituximab, cyclophosphamide, hydroxydaunorubicin hydrochloride (doxorubicin hydrochloride), vincristine (Oncovin), and prednisone therapy. According to Barr et al., R-CHOP therapy was associated with overall survival of 96% and 94% at three and five years, respectively [4]. One issue, however, is that there were many discontinuations of the maintenance therapy with rituximab [4]. The majority of discontinuations were due to acquired infections and the patient's decision to stop [4]. This therapeutic option is extremely effective in patients with follicular lymphoma and has been shown to reduce malignant cells within ascitic fluid if given through the intraperitoneal route [6]. Another standard treatment for advanced follicular lymphoma is bendamustine plus obinutuzumab-based chemotherapy [11]. Obinutuzumab therapy has been shown to increase progression-free survival over rituximab-based therapy, however, obinutuzumab had more adverse effects in clinical trials [11]. This becomes an issue because many patients cannot tolerate the full treatment course [4]. Treatment plans need to be further investigated and tailored to the individual patient in order to minimize secondary infections and non-compliance. Our patient underwent six cycles of R-CHOP therapy every three weeks in an outpatient setting.

In our patient, the triglyceride level was approximately 993 mg/dl. This patient had a chylous type of ascitic fluid due to follicular lymphoma. As touched on previously, it is believed that the pathophysiology is due to impaired lymphatic flow and may even involve direct invasion into the lymph system by the follicular lymphoma [1]. It has also been proposed that in NHL, as opposed to Hodgkin’s lymphoma, there is direct pleural infiltration rather than pathologic flow at the level of the thoracic duct [12]. The addition of pleural or peritoneal effusion in the case of an underlying lymphoma usually leads to a poorer prognosis and increased rates of relapse after treatment with chemotherapy [1]. Although effusions may be a common result of lymphoma, a chylous effusion is extremely rare. Treating its underlying cause, in this case, follicular lymphoma is crucial in helping resolve the effusion [1]. Part of the issue is that the treatment plan for follicular lymphoma is highly variable depending on the presentation and some patients may not even require treatment for a few years due to a lack of symptoms [1]. According to Bezbaruah, conservative measures can be taken such as dietary modifications to help reduce the chyle flow [1]. Such dietary modifications include eating a protein-rich, low-lipid diet with an emphasis on consuming more medium-chain triglycerides and less long-chain fatty acids, as they rely on chylomicrons within the intestinal lymphatics [1]. These dietary modifications may not always help the patient’s ascites, especially if symptoms of lymphoma are present (i.e. lymphadenopathy) [1]. At that point, emphasis should be placed on chemotherapy and/or radiotherapy, and even surgery to create a pleuroperitoneal shunt for refractory cases [1].

## Conclusions

This case explores the unique nature of how follicular lymphoma may present as opposed to more textbook examples. Our patient’s initial clinical picture was somewhat unclear but quickly resolved with advanced imaging, tumor markers, biopsy results, and specialist input. Prompt multidisciplinary workup and management, along with a consistent follow-up, have allowed for positive outcomes. This case emphasizes how a follicular lymphoma may present with rapid chylous ascites that can lead physicians to believe that the underlying pathology may be of gastroenteric origins. Although uncommon, this presentation of follicular lymphoma should be considered a 'must-not-miss' diagnosis and should be ruled in or out promptly with a high index of suspicion.
